# Mapping the Road Ahead: A Collective Journey in Artificial Intelligence for Health and Medicine

**DOI:** 10.3390/diagnostics16040544

**Published:** 2026-02-12

**Authors:** Daniele Giansanti

**Affiliations:** Centre IATIS, Istituto Superiore di Sanità, 00161 Rome, Italy; daniele.giansanti@iss.it or gianslele@gmail.com; Tel.:+39-06-49902701

Artificial intelligence (AI) has emerged as one of the most disruptive technologies in modern healthcare, particularly in the domain of medical diagnostics, where complex data streams, high-dimensional imaging, and heterogeneous clinical information increasingly challenge traditional analytical approaches [1,2]. In the past decade, AI systems powered by machine learning and deep learning have enabled the automated detection of subtle patterns in medical images, improved interpretation of laboratory and genomic data, and supported clinical decision-making processes that were previously labor-intensive, time-consuming, or subjective [3,4]. These advances have translated into improvements in diagnostic accuracy, workflow efficiency, and early disease detection, with significant implications for patient stratification and personalized medicine [5].

Among diagnostic domains, medical imaging remains the most mature and widely investigated area for AI adoption. In radiology and pathology, deep learning models have demonstrated strong performance in image classification, segmentation, and anomaly detection across multiple modalities, including computed tomography, magnetic resonance imaging, ultrasound, and whole-slide pathology images [6,7]. Beyond imaging, AI has shown growing potential in laboratory medicine, where algorithms assist in the interpretation of complex biochemical and hematological data, as well as in genomics and molecular diagnostics, supporting variant classification and biomarker discovery [8].

AI-driven diagnostic approaches are also gaining relevance in oncology, cardiology, and neurology, where multimodal data integration—combining imaging, clinical variables, and omics data—offers new opportunities for early detection, risk stratification, and prognosis estimation [5,9]. In these contexts, AI systems can assist clinicians in identifying clinically meaningful patterns that may not be readily apparent through conventional analysis, thus enhancing diagnostic confidence and decision support.

Despite these promising developments, fundamental challenges persist in the translation of AI innovations into routine clinical practice. Many diagnostic AI tools demonstrate enhanced performance in controlled research settings, yet evidence of consistent benefits on patient-level outcomes, such as reduced morbidity or mortality, remains limited. Moreover, barriers related to model explainability, data representativeness, bias, and robust external validation continue to hinder large-scale clinical adoption [2,9].

The contributions assembled in this Special Issue, “Artificial Intelligence for Health and Medicine,” [10] reflect the breadth and depth of current research at the intersection of AI and diagnostics, highlighting not only technical innovations and performance improvements, but also emerging strategies for explainable AI, multimodal diagnostic integration, and rigorous clinical evaluation, which are essential for the safe and effective implementation of AI-based diagnostic tools in healthcare.

The project has brought together a total of 18 contributions, in addition to the present introductory editorial. Overall, the Special Issue comprises 13 original research articles (Contributions 1–13) and 5 review papers, including 4 narrative reviews (Contributions 14–17) and 1 systematic review (Contribution 18).

Artificial Intelligence (AI) has demonstrated transformative potential across multiple diagnostic domains in healthcare, enabling improved accuracy, efficiency, and personalization of patient care. The original research articles (Contributions 1–13) in this Special Issue highlight two major trends: AI applied to imaging and radiology, where hybrid models, deep learning, and neuro-symbolic reasoning enhance diagnostic precision and interpretability; and AI applied to clinical and predictive data, supporting risk assessment, laboratory management, prenatal screening, symptom forecasting, and monitoring in high-intensity environments. Overall, these studies illustrate the capacity of AI to optimize clinical workflows, reduce redundant testing, and enable data-driven, patient-centered decision-making, while also highlighting ongoing challenges such as model explainability, generalizability, and clinical validation.

Across the thirteen original research articles in this Special Issue, AI demonstrates its versatility and impact across diverse diagnostic domains. Contribution 1 (Buican et al.) initiates this journey by developing an Integrated Pulmonary Severity Score (IPSS) for COPD, merging clinical and psychological factors such as anxiety and depression with explainable machine learning to refine symptom and prognosis prediction beyond traditional FEV1 measures. Building on the theme of interpretable AI in imaging, Contribution 2 (Almughamisi et al.) introduces an anatomy-guided hybrid CNN–ViT model with neuro-symbolic reasoning for early, multilabel diagnosis of thoracic diseases, ensuring clinical plausibility alongside high accuracy. Contribution 3 (Castilla et al.) translates AI from theory to practice, externally validating a triaging system for chest X-rays that effectively prioritizes abnormal findings and mitigates reporting backlogs in emergency care, while Contribution 4 (Özel et al.) applies deep learning to lateral cephalometric radiographs, improving orthodontic assessment of vertical dimensions and supporting individualized treatment planning.

Extending beyond imaging, Contribution 5 (Lohaj et al.) harnesses machine learning and explainable AI to analyze COVID-19 medical records, uncovering patterns that aid in understanding disease progression and guiding clinical decisions. Contribution 6 (Shuchami et al.) tackles workflow efficiency in pediatric emergency departments, leveraging AI to reduce redundant laboratory tests while safeguarding diagnostic quality and patient comfort. Contribution 7 (Ucan et al.) demonstrates AI’s power in automating radiology reporting through a Swin Enhanced Yield Transformer model for Turkish chest X-ray reports, transforming complex image data into actionable text for clinicians.

In prenatal care, Contribution 8 (Yalçın et al.) employs a hybrid AI approach to improve first-trimester Down Syndrome risk prediction, enhancing accuracy and reliability of early screening. In pediatrics, Contribution 9 (Turki & Turki) identifies phonological biomarkers for speech sound disorders in Saudi Arabic-speaking children, applying machine learning to elevate diagnostic precision. In interventional procedures, Contribution 10 (Ozkara et al.) uses AI to predict early outcomes of prostatic artery embolization, supporting personalized treatment decisions. Contribution 11 (Giarnieri et al.) focuses on cytology, creating user-friendly AI models that streamline pleural effusion analysis and empower clinicians facing complex malignant cases. Contribution 12 (Finkelstein et al.) brings AI to oncology symptom management, forecasting physical and mental deterioration during chemotherapy to enable proactive interventions. Finally, Contribution 13 (Mihai et al.) expands AI’s reach into operational medicine by developing the IIRPM score to monitor oxidative stress and inflammation in military personnel, illustrating its potential for health surveillance in high-intensity environments.

Shifting focus from original research to comprehensive analyses, Contributions 14–18 illustrate how AI is being synthesized across diagnostic domains, offering both critical evaluation and forward-looking perspectives. These reviews explore the application of AI in musculoskeletal, respiratory, oncologic, and reproductive health, highlighting methodological advances, practical challenges, and emerging opportunities to enhance diagnostic accuracy and personalized care.

Contribution 14 (Mercurio et al.) provides a comprehensive review of artificial intelligence for the diagnosis and management of patellofemoral instability, addressing the complex interplay of anatomical and biomechanical factors and demonstrating how machine learning and deep learning approaches can improve diagnostic precision and support individualized treatment planning. Contribution 15 (Sait & Shaikh) focuses on AI-powered detection techniques for chronic obstructive pulmonary disease, summarizing current methodologies, their potential to enhance early diagnosis, and the role of AI in overcoming limitations of traditional clinical tools. Contribution 16 (Lastrucci et al.) offers a narrative review of AI applications in breast imaging, synthesizing systematic evidence on mammographic practice and emphasizing opportunities to optimize screening, improve diagnostic accuracy, and integrate AI into routine clinical workflows. Contribution 17 (Moustakli et al.) examines AI in the assessment of reproductive aging, highlighting the influence of mitochondria, oxidative stress, and telomere biology, and discussing how machine learning can aid in predicting reproductive potential and informing patient counseling. Finally, Contribution 18 (Xu et al.) presents a systematic literature review of advanced deep learning approaches for comprehensive breast cancer assessment using whole slide images, demonstrating the capability of AI to enhance early detection of lymphocytes and molecular biomarkers, thus supporting precision diagnostics and improved patient outcomes.

Together, these eighteen contributions form a cohesive narrative of AI advancing diagnostics, from imaging to predictive analytics, and from workflow optimization to individualized patient care, highlighting both the opportunities and the challenges that define this rapidly evolving field.

Looking toward the future, the eighteen contributions in this Special Issue collectively map the evolving landscape of AI in medical diagnostics, demonstrating its transformative potential across clinical imaging, predictive analytics, workflow optimization, and patient-centered care. Moving forward, several key directions emerge.

*First*, the integration of multimodal data—combining imaging, laboratory, genomic, and patient-reported information—promises to enhance diagnostic precision and support holistic, individualized assessments (Contributions 1–18). Both the original research articles (Contributions 1–13) and the review studies (Contributions 14–18) emphasize the importance of interpretability and transparency, underscoring that clinically adopted AI systems must be explainable, reliable, and aligned with physiological knowledge to gain clinician trust.

*Second*, innovative model architectures, including hybrid networks, neuro-symbolic reasoning, and transformer-based frameworks, illustrate how AI can merge expert knowledge with algorithmic efficiency, improving generalizability and reducing bias (Contributions 2, 7, 8, 14–18). While the original research articles introduce and validate these architectures in experimental or clinical contexts, the review studies analyze and synthesize such approaches across multiple diagnostic domains, providing guidance for future model development.

*Third*, beyond imaging, AI’s predictive and monitoring applications highlight its expanding role in proactive and preventative healthcare. Predictive monitoring includes prenatal screening, pediatric speech diagnostics, and oncology symptom forecasting (Contributions 8, 9, 12). AI systems for workflow optimization and decision support, such as repeated lab testing in pediatric emergency departments and automated X-ray report generation (Contributions 6 and 7), enhance efficiency and streamline clinical processes. Contribution 7—focusing on automated X-ray report generation—illustrates how AI can optimize clinical workflows by producing structured, actionable text from images, complementing predictive monitoring systems without being predictive itself.
